# Applying the British Columbia Health System Matrix (BCHSM) population segmentation framework to support integrated care in Ontario, Canada.

**DOI:** 10.23889/ijpds.v7i3.1788

**Published:** 2022-08-25

**Authors:** Ruth Hall, Luke Mondor, Walter Wodchis

**Affiliations:** 1 HSPN

## Objectives

To adapt the BCHSM population segmentation methodology to Ontario’s health administrative data to identify mutually exclusive segments with similar health care needs to support integrated care efforts and population health management in Ontario, Canada. To compare health system related costs across derived segments to identify opportunities for better integrated care.

## Approach

We identified Ontarians alive with valid health card numbers as of April 1, 2020 (n =14,358,565) and created a matrix of prior utilization, cost and diagnoses using linked health administrative databases. Using a hierarchical technique, we assigned individuals into one of 14 BCHSM segments based on the greatest health care needs. Segments of need range from non-users (low need) to end-of-life patients (greatest need). We report the distribution of individual characteristics, average monthly costs across segments and further stratified health care costs by quintile of material deprivation within segments.

## Results

The largest segment was the healthy (low) users (43%) followed by low chronic conditions (28%) and non-users (10%). Five segments comprised <1% of the total population: end-of-life, frail in care, cancer, frail in the community and child and youth major. Average costs per month alive increased from $28 for the non-user segment to $5,100 for the end-of-life segment (0.5% of the population). Costs in the Frail with high chronic conditions segment ($2,740/mo) were 3-times higher than costs in the high chronic conditions segment ($930/mo), 6-times higher than costs in the medium chronic conditions segment ($450/mo), and 14-times higher than costs in the low chronic conditions segment ($193/mo). Results were generally more favourable in areas of low (vs high) material deprivation overall and within population segments.

## Conclusion

Using Ontario’s linkable health administrative data we have created an Ontario adaptation of the BCHSM needs-based population segmentation approach. Segmentation supports population health management as well as helping identify opportunities for improvement to strengthen integrated care and potential cost savings.

